# Effect of Mineral Composition and w/c Ratios to the Growth of AFt during Cement Hydration by In-Situ Powder X-ray Diffraction Analysis

**DOI:** 10.3390/ma13214963

**Published:** 2020-11-04

**Authors:** Bo Chen, Yongming Zhang, Qing Chen, Fei Yang, Xianping Liu, Jianguo Wu, Peiming Wang

**Affiliations:** 1 School of Materials Science and Engineering, Tongji University, Shanghai 201804, China; bo.chen@tongji.edu.cn (B.C.); zym126@tongji.edu.cn (Y.Z.); 1610413@tongji.edu.cn (F.Y.); lxp@tongji.edu.cn (X.L.); wjg@tongji.edu.cn (J.W.); tjwpm@126.com (P.W.); 2 Key Laboratory of Advanced Civil Engineering Materials, Tongji University, Ministry of Education, Shanghai 201804, China

**Keywords:** AFt (Ettringite) Growth, cement hydration, mineral composition, in-situ investigation, Powder X-ray diffraction (XRD)

## Abstract

AFt is one of the major products at the early stage of cement hydration. It is an important product that influences the performance of the fresh and hardened cement pastes such as the setting time. However, there is a lack of detailed investigation on the growth of AFt in the cement pastes with a long-time scale. In this work, we reported a detailed analysis by using in-situ powder X-ray diffraction (XRD) on the growth of AFt in the cement pastes during hydration. Samples of the hydrated ordinary Portland cement (OPC) and another locally produced Portland cement with very high tricalcium silicate (C_3_S) content with different water–cement (w/c) ratios were investigated continually till they were hydrated for about 270 days by powder XRD. The work shows that during Portland cement hydration, the AFt reaches its maximum content with very high speed within about 24 h, which is influenced by the content of C_3_S in the raw cement samples and the w/c ratios of the cement pastes. Once the maximum content of AFt was reached, it decreases very fast within the following couple of days, and then decreases slowly and finally reaches a stable level at the late stage of hydration. The results also present that a lower w/c ratio is beneficial to the formation of AFt and the conversion of AFt to AFm as well. While higher w/c ratios are favorable for the AFt to remain stable in the hardened cement pastes.

## 1. Introduction

AFt, the most important phase of which is ettringite, is one of the major determinants of the hydration properties and performance of Portland cement at early stages such as the initial setting time [1] and the early strength [2] of the hardened cement pastes. It also influences the long-term service performance of the cement concrete such as the premature concrete deterioration, which is related to the secondary or delayed AFt formation [3,4,5]. AFt is also a major product generated from the hydration of calcium sulphoaluminate cement, expansive cement and rapid hardening cement [6,7,8].

Researchers have extensively studied the formation conditions and mechanism of AFt [9,10,11,12] and its structures [13,14,15,16]. It is found that AFt forms as long as there is the existence of gypsum, i.e., the presence of sulfate ions, in the cement pastes [17]. Once the sulfate ions are depleted, AFt would start to transform into monosulfoaluminate (AFm) by reacting with the remaining tricalcium aluminate (C_3_A) in the cement pastes [18]. Studies show that AFt has the same chemical composition and the same crystal structure while formed during the hardening of cement concrete, although its crystals could present a small change in lattice parameters such as an increase of the a lattice parameter and a decrease of the c lattice parameter by the effects from external conditions, but without any crystal structural modification [15]. However, the sizes and morphology of the AFt crystals could be varied from case to case [19,20]. Except for the typical needle-shaped crystals [19,21], AFt could also be spherical [22], cubic-shaped [19] or in small massive clusters [3,23]. Most of these studies are focusing on the AFt formation mechanism or structure investigation [24]. Few of them are quantitative or semi-quantitative analyses on the growth of AFt during the hydration and hardening of cement pastes, especially at long-time scales (over six months of curing). Clearly, quantitative or semi-quantitative characterization [11,25,26] of the growth and development of AFt in the cement pastes will enrich the cement hydration mechanism.

In this research, the hydrated pastes of an ordinary Portland cement (hereinafter simplified as OPC) and a locally-produced Portland cement with very high tricalcium silicate (C_3_S) content (hereinafter simplified as HCPC (for high C_3_S Portland cement)) mixed at two different water–cement (w/c) ratios are studied by powder X-ray diffraction (XRD). Among which, the HCPC is a high-performance Portland cement, which could work with a higher amount of industrial waste admixture such as fly ash and coal gangue, hence could reduce the total energy consumption and the environmental pollution of the cement industry. In this work, the development of the AFt during the hydration of these cements was recorded and analyzed in a native state to understand the influence of C_3_S content to the formation of AFt. It also provides experimental proof to enrich the formation and growth mechanism of AFt during cement hydration.

## 2. Experiments

### 2.1. Materials

The used OPC is a commercial OPC manufactured by Anhui Conch Group Co. Ltd., Anhui, China; the HCPC is produced locally in a laboratory. The measured chemical composition of the two raw cement samples, by X-ray fluorescent (XRF) method (Bruker AXS SRS3400 machine was used, Bruker Corporation, Berlin, Germany), are shown in Table 1. The main mineral compositions of the two cements, obtained by the Rietveld refinement analysis, are shown in Table 2. The details of the Rietveld refinement analysis are presented in Figures S1 and S2 and Tables S1 and S2 in part 1 of the supplementary information.

### 2.2. Methods

The powders of two cements (HCPC and OPC) were mixed with deionized water at two different w/c ratios of 0.5 and 0.3. Once the mixing of each sample was finished, the fresh cement pastes were immediately sealed in the standard glassy diffractometer sample holders. These 4 cement pastes were kept for the repeated in-situ powder XRD measurements in the future. A dedicated X-ray transparent film with a thickness of 2.5 μm, MYLAR X-ray film (Cat. No. 100) produced by Chemplex Industries Inc. (Palm City, FL, USA), was used to seal the samples. The used X-ray film was verified having almost no influence on the AFt’s characteristic peak for powder XRD measurements (see Figure S3 in the Supplementary Information). The sealing would reduce the measurement errors caused by water evaporation of the hydrating cement pastes and difference of the measured specimen regions [27,28]. It also reduces carbonation of the hydrated cement pastes and avoids doing the stopping-hydration treatment to the cement paste samples during experiments (while they are hydrating and hardening).

During the experiments, the environment temperature was maintained at 20 ± 1 ℃, and the relative humidity was maintained at 50 ± 5%. The cement pastes were cured in this environment and the XRD measurements were carried out in this environment as well.

A powder X-ray diffractometer (Rigaku D/max 2550, Rigaku Corporation, Tokyo, Japan) with Cu Kα radiation of 0.154 nm wavelength (X-ray energy of 8.04 keV) was used for measurements. Since the (2θ) angle of the characteristic peak of AFt crystals under the Cu Kα X-ray illumination equals about 9.0°, the samples were measured by stepping scans in the 2θ range of 8.5°–9.5° and were monitored by fast continuous scans in the 2θ range of 5°–50°. All the stepping scans were performed with a working voltage of 40 kV, an operating current of 250 mA, a step size of 0.02° and an exposure time of 4 s/step. All the continuous scans were performed with a working voltage of 40 kV, an operating current of 250 mA and a scanning speed at 10°/min.

For all the 4 sealed cement paste samples, the same region of each sample was measured by XRD, in the native state, once they reached the targeted hydration times during hydration. The growth of AFt during the cement hydration was recorded by continually measuring the XRD patterns of these samples between 8.5° and 9.5°. According to the principle that, under the same powder XRD measurement conditions, the change of the integral intensities of the same diffraction peaks of a crystalline phase is proportional to the change of the content of this phase in the sample [29], the development of the AFt crystalline phase in the samples was monitored.

## 3. Results and Discussions

### 3.1. XRD Patterns of the Hydrated Samples

**Figure 1:**
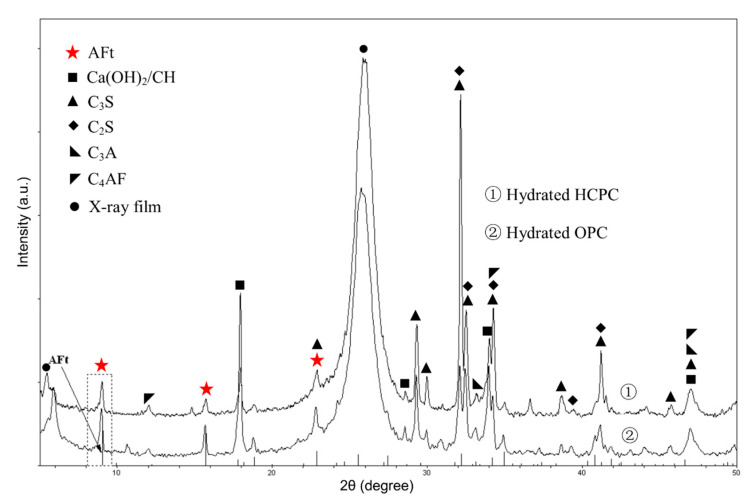
XRD patterns of the hydrated HCPC and OPC after 23 h.

**Figure 2:**
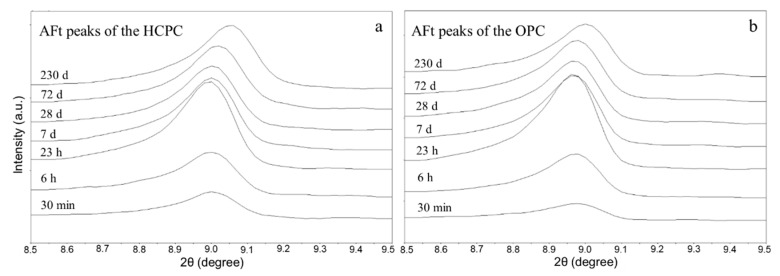
Stepping scan patterns of the AFt’s characteristic peak of the HCPC (**a**) and the OPC (**b**) at different hydration ages.

Figure 1 shows the XRD patterns of the hydrated HCPC and OPC at the w/c ratio of 0.5. The stepping scan results of the characteristic peaks of AFt (8.5°–9.5°) in both hydrated cement pastes at some of the chosen hydration ages are given in Figure 2.

These patterns indicate that after 23 h of hydration, the area of AFt’s characteristic peaks increased significantly compared with that of at the very beginning. This means that a large amount of AFt was produced after 23 h.

### 3.2. Comparison Analysis of the Integral Intensities of the Diffraction Peak of AFt

Under the same measurement conditions, the change of the integral intensities of the same X-ray diffraction peak of the same component determines the change of its content or proportion in the whole sample, in case the crystal structure of this component remains constant.

**Figure 3:**
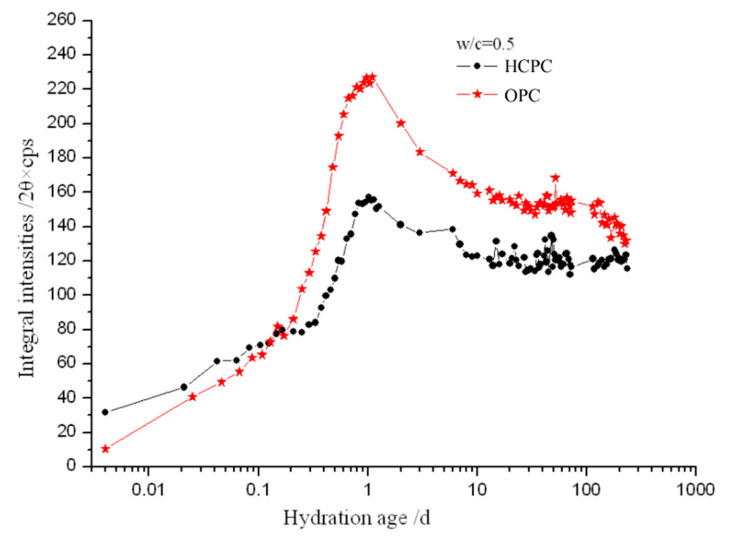
The development of the integral intensities of the AFt’s characteristic diffraction peak in the HCPC and OPC with w/c = 0.5.

**Figure 4:**
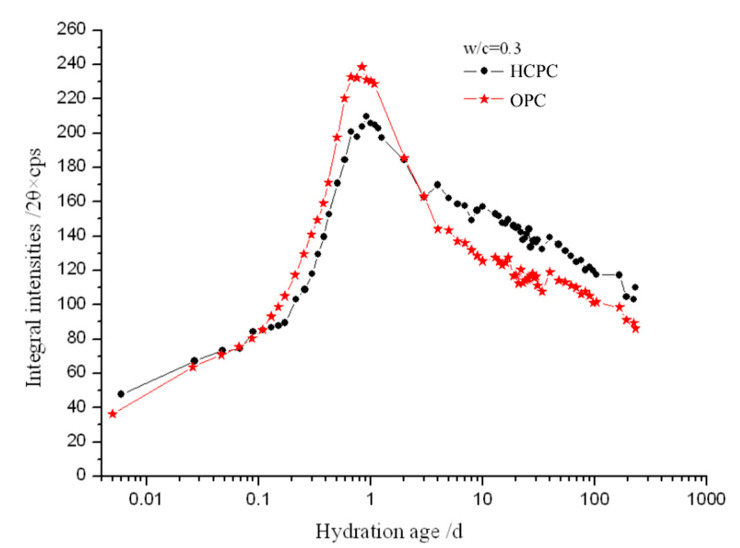
The development of the integral intensities of the AFt’s characteristic diffraction peak in the hydrated HCPC and OPC with w/c = 0.3.

In our work, the characteristic XRD peak of the AFt crystal was recorded at the different hydration times in the native state, and the integral intensities of the recorded peaks were calculated. Figure 3 and Figure 4 are the comparison of the development of the integral intensities of the AFt’s characteristic peaks in the HCPC and OPC pastes at different hydration times and with different w/c ratios of 0.5 and 0.3.

It can be seen from both Figure 3 and Figure 4 that AFt is produced very shortly after the cement specimens were mixed with water, i.e., about a few minutes after mixing. The AFt content increased rapidly in the cement pastes at the early time of the hydration, which indicates that the C_3_A mineral and gypsum in the cement reacted rapidly after mixing with water and the AFt crystals precipitated out immediately. The content of AFt reaches the maximum within about 24 h, closer to 23 h, in all the hydrated cement pastes after a rapid increase. Then the AFt content starts to decrease. Clearly, the decrease of AFt content was caused by the depletion of available solid gypsum in the system after a while of cement hydration, the CaSO_4_ starts to remove from the solution quickly, [Ca^2+^] and [SO_4_^2^^−^] declined sharply, the generated AFt crystals began to react with calcium aluminate (hydrates) to form mono-sulfate calcium sulphoaluminate hydrate (AFm) [1], so the content of AFt continues to reduce while the cement hydration goes further on.

It also can be seen that, with the same w/c ratio, in a very short period of time (no longer than 3 h) after the beginning of hydration reaction, there is more AFt produced in the HCPC than in the OPC pastes. Then the content of AFt produced by OPC pastes increased, and its AFt content becomes more than that in the HCPC pastes until at least a couple of days later. This is because, at the beginning of hydration, the HCPC can provide more [Ca^2+^] for the reactions due to the higher total content of C_3_A and C_3_S, which made the concentration of [Ca^2+^] in the liquid phase in the HCPC pastes higher than that of in the OPC pastes. The HCPC also contains more gypsum, which is conducive to the formation of AFt. These made the hydrated HCPC produce more AFt than the hydrated OPC at the beginning of the hydration. Then, due to the saturation of [Ca^2+^] and [SO_4_^2-^] in the liquid phase, there is a large amount of Ca(OH)_2_ (simplified as CH, see the CH’s characteristic diffraction peak at about 18° in Figure 1) produced in the HCPC paste, which inhibits the dissolution of AlO_2_^−^ into the liquid phase and slows down the hydration of C_3_A, thus reduces the formation speed of AFt. Additionally, AFt may also be formed through topochemical reaction in OPC, since alumina dissolved from the pozzolans in the OPC is more reactive than those from C_3_A [30]. This makes the amount of AFt produced in the hydrated OPC pastes catch up that of in the hydrated HCPC pastes after a short time, and then overtake it. The overtaking point happened at different times in the samples with different w/c ratios. The overtaking point for the hydrated OPC paste with w/c ratio of 0.5 is at around 3 h, which happens about 1.5 h later than the sample with w/c ratio of 0.3, the overtaking point of which was at around 1.5 h. This is because when the w/c ratio is lower, the ion concentration in the liquid phase in the cement pastes can be saturated in a shorter time; thus, for the hydrated HCPC, the advantage of having higher concentration of [Ca^2+^] in the liquid phase to produce AFt at the very beginning of hydration would be extinguished sooner. Furthermore, the large amount of CH produced by hydration and the unreacted gypsum would slow down the hydration of C_3_A in the HCPC paste significantly [31]. At the same time, with lower w/c ratio, the concentration of alkali, NaOH and KOH, in the hydrated OPC pastes would be even higher, which will accelerate the hydration of C_3_A [32]. All of these reasons resulted in that the content of AFt in the hydrated OPC catches up the content of AFt in the hydrated HCPC in a shorter time if the w/c ratio of the cement pastes was lower.

Similar to the above, at the w/c = 0.5, time for the content of AFt to reach the maximum in the cement pastes is also about 1.5 h later than that in the cement pastes with the w/c = 0.3. The former one reaches the maximum in about 24 h, and the latter one is in about 22.5 h. This is because when the w/c ratio is lower, NaOH and KOH in the liquid phase of the cement paste could reach higher concentration quicker, then the hydration of C_3_A could be faster, which accelerated the formation of AFt. In addition, both a topochemical reaction [33] and through-solution reaction are carried out with all the necessary reactive materials in water. When the w/c ratio is low, it is also possible to produce AFt faster by topochemical and through-solution reactions due to the extra high concentration of those necessary ions, including Ca^2+^, which further accelerates the consumption of gypsum and makes the production of AFt reach the maximum earlier. It can also be seen that although the content of CaSO_4_ in the OPC is slightly lower than it is in the HCPC (the relative ratio is 25:27), the maximum production of AFt from the OPC is higher than that of the HCPC. This is mainly because the relative amount of C_3_A to CaSO_4_ of the OPC (32:25) is higher than that of HCPC (20:27). According to the reaction for producing AFt:

$$C_{3}{A+3C\bar{S}H}_{2}+26H\to C_{6}{A\bar{S}}_{3}H_{32}$$

The higher relative amount of C_3_A to CaSO_4_ will benefit the formation of AFt according to chemical kinetics. Secondly, when the content of gypsum is less than 20%, C_3_S may react with C_3_A to produce aluminosilicate hydrate [18]. Thus, HCPC will consume more C_3_A due to higher content of C_3_S, and if the content of CaSO_4_ were at a similar level, OPC will produce more AFt at last.

**Figure 5:**
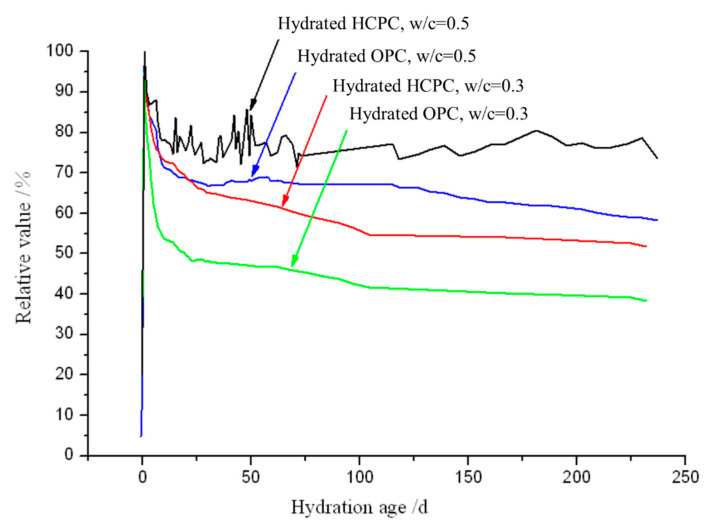
Profiles of the percentages of the integral intensities of the AFt’s characteristic peaks, at different hydration ages, relative to the reached maximum intensities of the AFt peak of the samples.

Figure 3 and Figure 4 also show that once the content of AFt reaches the maximum at about 24 h, it starts to decrease immediately, and the decreasing speed is very quick. Also, the content of AFt in the hydrated OPC decreases much faster than in the HCPC pastes (see Figure 5 as well) till about 10 days. For the hydrated OPC with w/c ratios of 0.5 and 0.3, the integral intensities of the AFt’s characteristic peak decreased to 70% and 52%, respectively, after 10 days of hydration, compared with the maximum values reached. While for the hydrated HCPC, the integral intensities of the AFt’s characteristic peak decreased to 78% and 74% of the maximum, respectively, under the same conditions. This is because there is higher concentration of NaOH and KOH in the liquid phase of the hydrated OPC, and this will accelerate the hydration of C_3_A, thus increasing the conversion of AFt to AFm after the available solid-state gypsum has been fully consumed. In addition, the content of CaSO_4_ in the OPC is a bit lower, compared with the HCPC’s, thus its capability to maintain the AFt is weaker. Therefore, after reaching the maximum values, the decreasing rate of the AFt content in the hydrated OPC is faster than that in the hydrated HCPC till about 10 days. With a w/c = 0.3, the same above reasons also resulted in a decrease of the AFt content in the hydrated OPC to a level comparable to that of in the hydrated HCPC after two days of hydration, and the AFt content in the hydrated OPC further decreased to a level lower than that of in the hydrated HCPC only after three days of hydration. While the w/c = 0.5, the AFt content in the hydrated OPC was only decreased to the level closing to that of the hydrated HCPC, but did not decrease to the level lower than that of the hydrated HCPC even after over 200 days. This is because when the w/c ratio is relatively low, the concentrations of NaOH and KOH in the hydrated OPC paste are relatively high, hence the C_3_A’s hydration within them were accelerated heavier and the consumption of CaSO_4_, to form AFt, is relatively high in the early stage of the hydration. This then weakens the delaying effect of gypsum and CH to C_3_A’s hydration compared with higher w/c ratio cases, which resulted in that the fastest decreasing rate of the AFt content happened in the hydrated OPC paste with the lowest w/c ratio.

Figure 5 shows that the AFt content in all the hydrated samples are almost rocketing to the maximum values. This indicates that the AFt is formed with an extremely fast reaction process. Once the content of AFt reach maximum, they start to decrease right away, and the rate of decrease is slow compared with their growth. Especially after 30 days of hydration, the decreasing rate is very slow, indicating that the conversion of AFt to AFm is a very slow process.

**Figure 6:**
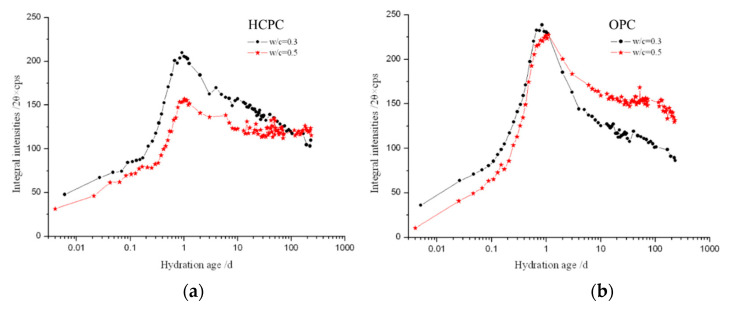
Plot of the integral intensities of the AFt’s diffraction peak of the HCPC (**a**) of the OPC (**b**).

Interestingly, for the samples with w/c = 0.5, the continuous decrease of the AFt content stopped after about 30 days of hydration. Since then, the development of AFt content during hydration of the HCPC and the OPC are quite different. In the hydrated HCPC, the AFt content goes up and down after 30 days (see the black line in Figure 3 as well), and then gradually decreased and stabilized after another 20 days. It can be seen from Figure 6a that the maximum content of AFt produced by the hydrated HCPC with the w/c = 0.5 is significantly lower than the sample with the w/c = 0.3. The maximum integral intensity of the AFt’s characteristic peak from the former specimen is only about 75% of the latter one (see Figure 6a). This could be caused by that fact that the total concentration of NaOH and KOH, i.e., the total concentration of OH^-^, is lower in the HCPC paste with w/c = 0.5 compared with the case of w/c = 0.3 at the early time (within 24 h) of the hydration. Hence, the promotion to C_3_A’s hydration from the alkaline ions in the HCPC paste with w/c = 0.5 is strongly retarded by CH and gypsum, and thus prevented part of the C_3_A reacting with CaSO_4_ to produce AFt. This means that within the hydrated HCPC with w/c = 0.5, there was some gypsum remained after the produced AFt reached the maximum content. This phenomenon could be caused by the gradual conversion of AFt to AFm, in the following hydration, which destroyed the protective shell formed by the previously generated AFt on the surface of C_3_A. The loss of protective shell leads C_3_A to rehydrate and react with the remaining CaSO_4_ again, hence to produce AFt again. This makes the amount of AFt increase, re-shell and isolate the C_3_A from CaSO_4_ once again. Thus, AFt begins to convert into AFm again, destroys the protective shell formed on the surface of C_3_A again, which leads to rehydration of C_3_A, and reacts with CaSO_4_ to produce AFt. Hence, the content of AFt increases again. This cycle repeats several times in the specimen until all the gypsum is consumed. Then, AFt is no longer produced and slowly converts into AFm, which produces the above-mentioned fluctuating phenomenon, i.e., the AFt content in the hydrated HCPC with w/c = 0.5 goes up and down for over 20 days after 30 days of hydration, and then the content of AFt gradually decreases and keeps at a stable amount, which is about 75% of the maximum production of AFt.

While the hydrated OPC with w/c = 0.5 has a different process, the AFt content remains almost stable after 30 days of hydration and then begins to decline after about 130 days with a very slow rate. After 230 days of hydration, the AFt content decreases to 58% of the maximum from the 68% at 130 days of hydration. This is because AFt was converted into AFm, which gradually destroyed the protective shell formed by the precipitation of AFt on the surface of C_3_A particles as well. At the same time, the content of free water in the later age of hydration is greatly reduced and the total concentration of NaOH and KOH in the liquid phase of the specimen increases. Under the heavy alkaline environment (more than 0.5 N) [34] for a long-term, AFt starts to convert into AFm again, hence its content keeps decreasing.

Compared with the hydrated specimens with w/c = 0.5, in the specimens with w/c = 0.3, the AFt content keeps decreasing continuously after it reached the maximum value without stopping. However, their decreasing rates gradually reduce as well, and become extremely slow in the late stage of hydration. This obviously results from the gradual conversion of AFt to AFm under high concentrations of NaOH and KOH environments at low w/c ratio conditions.

Figure 6a,b show that, for the same cement, the amount of produced AFt at the early age of hydration (within 24 h) is higher in the specimens with w/c = 0.3 than in these with w/c = 0.5. This is because when the w/c ratio is lower, there is less free water in the specimens, the total concentration of NaOH and KOH is higher. At the same time, in the lower w/c ratio specimens, the generated AFt in the liquid phase would reach the saturation level quicker after C_3_A reacted with CaSO_4_, and it could then precipitate and form into crystals faster. This makes the samples with lower w/c ratio produce more AFt at the early age of hydration.

Figure 6 also presents that after the AFt content reached its maximum (at about 24 h), the decreasing rate of AFt content in the specimens with w/c = 0.3 is quicker than that with w/c = 0.5. Especially for the OPC, a short time after the AFt content of the specimen with w/c = 0.3 reached the maximum, i.e., after two days of hydration, its AFt content decreased to the level less than in the specimen with w/c = 0.5. This is also because the total concentration of NaOH and KOH in the liquid phases is higher in the hydrated cement paste with lower w/c ratio. While, after a long time, the AFt content in the hydrated HCPC with w/c = 0.3 only decreased to the level close to the specimen with w/c = 0.5. Even after 200 days of hydration, its AFt content is only slightly lower than that in the specimen with w/c = 0.5. This is mainly because the hydration of HCPC produced more CH, and it could work together with gypsum to significantly delay the hydration of C_3_A. Hence the AFt content in the hydrated HCPC decreases more slowly.

## 4. Conclusions

(1) AFt crystals form in a few minutes with an extremely fast reaction process after mixing the cement with water. The content of AFt in the cement pastes reaches the maximum within 24 h after cement hydration; it then gradually reduces and eventually maintains at a stable level.
(2) With comparable amount of CaSO_4_, the cement with higher C_3_S content, i.e., the HCPC, produces more AFt than the cement with lower C_3_S content such as the OPC, at the initial stage of hydration (within 2–4 h). However, the production of AFt in the cement with lower C_3_S content (OPC) becomes more than that in the cement with higher C_3_S content soon after the initial stage, and the highest production of AFt in the cement with lower C_3_S content (OPC) reaches a higher level. Yet, the AFt content keeps at a more stable level in the cement with higher C_3_S content during hydration.
(3) A lower water–cement ratio is beneficial to the formation of AFt and the conversion of AFt to AFm as well. On the other hand, i.e., at a higher water–cement ratio, it is favorable for the AFt to remain stable.
(4) It is possible to produce AFm during hydration even when there is the presence of gypsum in the specimens, and this remaining gypsum could also react with C_3_A as well to produce AFt in a higher C_3_A environment.

## Figures and Tables

**Table 1:** Chemical compositions of the two different cement specimens (wt.%).

Cement	CaO	SiO_2_	Al_2_O_3_	Fe_2_O_3_	SO_3_	MgO	K_2_O	Na_2_O	F-CaO
OPC	62.72	20.24	5.29	3.40	1.97	1.18	0.57	0.11	0.23
HCPC	64.78	20.34	5.02	3.11	2.20	1.09	0.35	0.10	0.10

**Table 2:** Mineral compositions of the two different cement specimens (wt.%).

Cement	C_3_S	C_2_S	C_3_A	C_4_AF	Gypsum
OPC	55.03	21.84	5.59	10.48	4.33
HCPC	68.02	10.47	3.39	13.37	4.75

## Supplementary Materials

The following are available online at https://www.mdpi.com/1996-1944/13/21/4963/s1, Figure S1: Rietveld refinement analysis of the raw OPC specimen, Table S1: Rietveld refinement analysis result of the raw OPC specimen, Figure S2: Rietveld refinement analysis of the raw HCPC specimen, Table S2: Rietveld refinement analysis result of the raw HCPC specimen, Figure S3: Verification XRD measurement on a hardened cement paste after 1 day of hydration.
