# Supplementary material for: Effect of Mineral Composition and w/c Ratios to the Growth of AFt during Cement Hydration by In-Situ Powder X-ray Diffraction Analysis
Source: Materials (Basel). 2020 Nov 4;13(21):4963. doi: 10.3390/ma13214963 (PMC7662245; doi:10.3390/ma13214963)
Supplement: Supplementary file 1 [file materials-13-04963-s001.pdf]

Effect of Mineral Composition and w/c Ratios to the Growth of AFt during Cement Hydration by In-Situ Powder X-Ray Diffraction Analysis

Bo Chen [1,2], Yongming Zhang [1,2], Qing Chen [1,2,*], Fei Yang [1], Xianping Liu [1,2], Jianguo Wu [1] and Peiming Wang [1,2]

[1] School of Materials Science and Engineering, Tongji University, Shanghai 201804, China; bo.chen@tongji.edu.cn (B.C.); zym126@tongji.edu.cn (Y.Z.); 1610413@tongji.edu.cn (F.Y.); lxp@tongji.edu.cn (X.L.); wjg@tongji.edu.cn (J.W.); tjwpm@126.com (P.W.)

[2] Key Laboratory of Advanced Civil Engineering Materials (Tongji University), Ministry of Education, Shanghai 201804, China

* correspondence: 15666022@tongji.edu.cn; Tel.: +86-21-39526230

1. XRD characterization of the raw cements

The powder of the 2 raw cement specimens, OPC and HCPC, were measured by the same powder X-ray diffractometer (Rigaku D/max 2550) with Cu Kα radiation of 0.154 nm wavelength (X-ray energy of 8.04 keV). The samples were measured by stepping scans in the 2θ range of 5°–90°. The scans were performed with a working voltage of 40 kV, an operating current of 250 mA, a step size of 0.02° and an exposure time of 4 s/step.

The obtained powder XRD patterns of the 2 raw cements were analyzed by Rietveld refinement method (see Figures S1 and S2), and the results are presented in Tables S1 and S2.

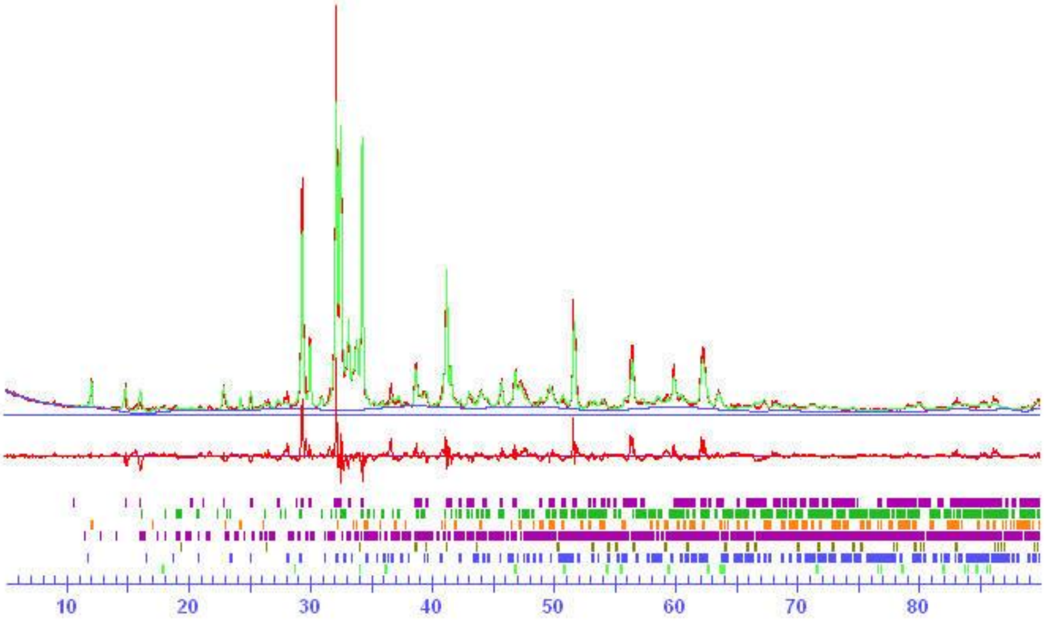

Figure S1. Rietveld refinement analysis of the raw OPC specimen.

Table S1. Rietveld refinement analysis result of the raw OPC specimen.

Phases	C_3S	C_2S	C_4AF	C_3A	Gypsum
wt.%	55.031	21.838	10.482	5.591	4.332
e.s.d (wt.%)	0.225	1.186	0.307	0.445	0.167
R Values	Rp=12.734, Rwp=17.140, Re=6.386, GoF=2.684				

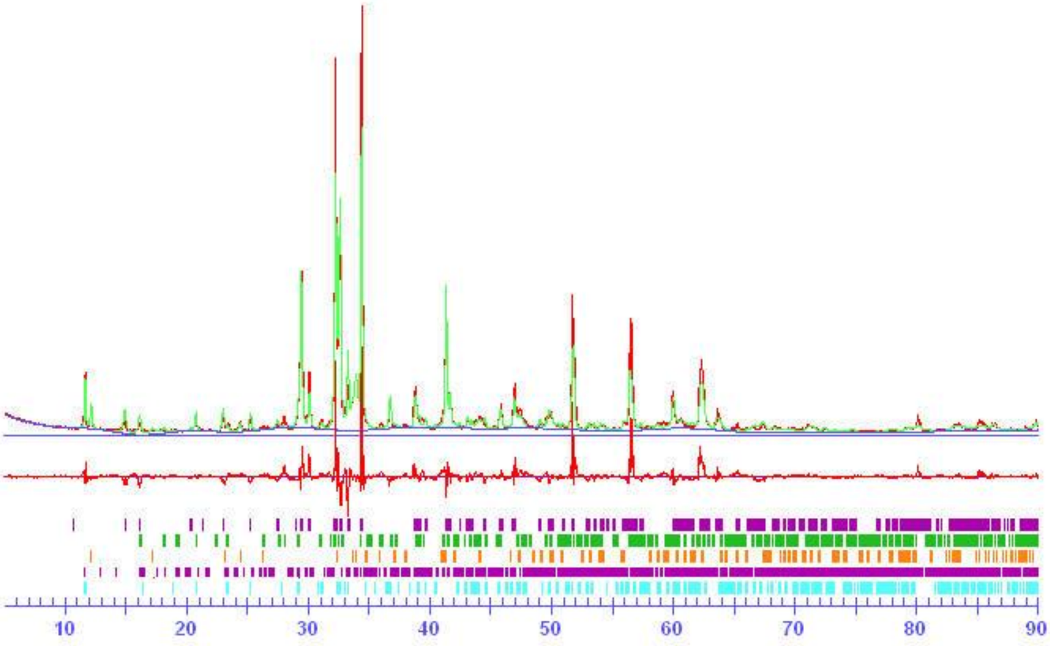

Figure S2. Rietveld refinement analysis of the raw HCPC specimen.

Table S2. Rietveld refinement analysis result of the raw HCPC specimen.

Phases	C_3S	C_2S	C_4AF	C_3A	Gypsum
wt.%	68.020	10.474	13.365	3.394	4.746
e.s.d (wt.%)	0.179	0.537	0.341	0.562	0.369
R Values	Rp=15.136, Rwp=20.132, Re=6.237, GoF=3.228				

2. Verification measurements on using the X-ray film for sealing

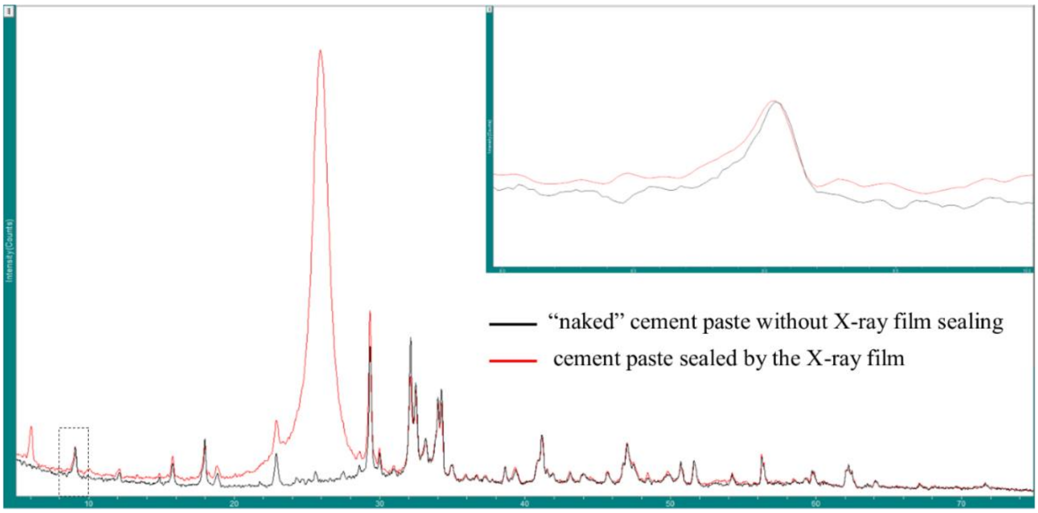

Figure S3. Verification XRD measurement on a hardened cement paste after 1 day of hydration.

A verification measurement on a hardened cement paste after 1 day of hydration was done. The mixed fresh (OPC) cement paste with w/c ratio of 0.5 was immediately sealed in the standard glassy diffractometer sample holder once the mixing was finished. The dedicated X-ray transparent film with a thickness of 2.5 μm, MYLAR X-ray film (Cat. No. 100) produced by Chemplex Industries Inc. (USA), was used to seal the sample. After 1 day of hydration, the hardened cement paste sealing by the X-ray film was measured by the powder X-ray diffractometer (result shown in the red line in Figure S1). Then the X-ray film was peeled off, and the "naked" sample was measured again (result shown in the black line in Figure S1). The results were shown in Figure S1, in which the upper-right inset is the zoomed-in XRD pattern of the sample within the 2θ range of 8.0°-10.0°. The AFt's characteristic peaks (in the 2θ range of 8.5°-9.5°) in both XRD patterns with and without the X-ray film are almost fully overlapped with each other (see Figure S1) which means the AFt peak in both measurements are almost the same and the used X-ray film almost have no influence on the AFt's characteristic peaks for powder XRD measurements.
